# The age of enlightenment in melanoma immunotherapy

**DOI:** 10.1186/s40425-018-0397-8

**Published:** 2018-08-22

**Authors:** Mark R. Albertini

**Affiliations:** 1 University of Wisconsin Carbone Cancer Center, Madison, WI USA; 2 Department of Medicine, University of Wisconsin School of Medicine and Public Health, Madison, WI USA; 3 Medical Service, William S. Middleton Memorial Veterans Hospital, Madison, WI USA; 4 University of Wisconsin Clinical Sciences Center, Room K6/530, 600 Highland Avenue, Madison, WI 53792 USA

**Keywords:** Metastatic melanoma, Immunotherapy, Immune-checkpoint blockade

## Abstract

An updated survival analysis by Callahan et al. published in the February 1, 2018 issue of the *Journal of Clinical Oncology* reported a 3-year overall survival (OS) rate of 63% for 94 patients with previously treated or untreated advanced melanoma who received ipilimumab and nivolumab as concurrent therapy in a phase 1 dose escalation study CA209–004 (*n* = 53) or in an expansion cohort with the dose and schedule of concurrent ipilimumab and nivolumab now approved for patients with unresectable or metastatic melanoma (*n* = 41). While this 3-year OS rate of 63% in patients with measurable, unresectable stage III or IV melanoma is an impressive accomplishment that compares very favorably with historical metastatic melanoma survival rates, findings from larger phase 3 studies are needed to determine whether combination immunotherapy significantly improves survival more than single agent immunotherapy with PD-1 blockade. This Commentary discusses the transition from the dark ages to the age of enlightenment in melanoma immunotherapy and provides a roadmap for a better tomorrow for patients with metastatic melanoma.

Unprecedented treatment advances for patients with unresectable stage III or stage IV melanoma have occurred in the past few years. Consensus guidelines are available for treating metastatic melanoma patients with immunotherapy options currently approved by the US Food and Drug Administration (FDA) including high-dose interleukin-2 (IL-2), ipilimumab, nivolumab, pembrolizumab, the combination of ipilimumab and nivolumab, and talimogene laherparepvec (T-VEC; for patients with accessible lesions) [1]. We now have report of a 3-year OS rate of 63% in 94 patients with measurable, unresectable stage III or IV melanoma who received ipilimumab and nivolumab as concurrent therapy [2]. In contrast, a 1-year OS rate of 25.5% was provided in a 2008 meta-analysis of 42 phase II cooperative group trials (70 trial arms) as a reference point for future phase II metastatic melanoma studies [3]. As phase 1 studies historically overestimate survival rates, the magnitude of recent advanced melanoma treatment progress is perhaps best appreciated by comparing treatment options for patients with unresectable stage III or stage IV melanoma in 2018 with treatment options for similar patients before the year 2011. The treatment options before 2011 included dacarbazine (DTIC), an alkylating agent approved by the US FDA in 1975 for the treatment of advanced melanoma [4]. The efficacy of DTIC in metastatic melanoma was low, without a confirmed survival benefit, and with a transient response realized in only 10–20% of patients. While many other chemotherapeutic agents were also tested in metastatic melanoma, none achieved a meaningful survival benefit over DTIC alone. Cytokine-based therapy with high dose bolus IL-2, pioneered by Dr. Steven Rosenberg (Chief, Surgery Branch and Head, Tumor Immunology Section; National Cancer Institute), provided insights about the host immune response to cancer and was shown to have an overall response rate of 16% and a 6% complete response rate in metastatic melanoma [4]. However, treatment with high dose IL-2 had severe toxicity and was limited to excellent performance status patients at specialized treatment centers with expertise in managing severe high dose IL-2 associated toxicities. While participation in an appropriate clinical trial offered a theoretical basis for clinical benefit, that benefit was not often achieved in the pre-immunotherapy era. The unmet need to successfully translate lab insights into the melanoma clinic for most metastatic melanoma patients, metaphorically speaking, represented a “dark ages” for treatment of advanced melanoma. The historical Dark Ages is generally considered to be a period of intellectual depression in the history of Europe during the Middle Ages. The “dark ages” of melanoma immunotherapy can be considered to include that period before the year 2011 when few effective systemic treatments were available in the clinic for metastatic melanoma patients. The prognosis was poor, medical management had limited impact on that poor prognosis, and outcomes were almost universally fatal. As summarized in a 2011 metastatic melanoma overview and update, “the standard of care for patients with metastatic melanoma has not changed significantly in the past 20 years and new strategies for treatment of metastatic melanoma are urgently needed” [4]. However, the beginning of a new age in melanoma therapy was also predicted as “significant insights have recently been gained into the molecular events underpinning the development of melanoma. A number of novel compounds designed to target these molecular events, as well as monoclonal antibodies to key immune regulatory functions, have been developed and used in clinical trials. The results of these trials hold great promise for the treatment of subsets of patients with metastatic melanoma” [4]. The identification of driver mutations and genetic aberrations in melanoma allowed for the development of therapies with drugs targeting the mitogen-activated protein kinase (MAPK) pathway (BRAF and MEK inhibitors). This translational insight has greatly advanced the care of metastatic melanoma patients with BRAF mutations and is reviewed elsewhere [5]. The evolution of practice-changing advances in melanoma immunotherapy are discussed here.

Compared to the “dark ages” (referring primarily to the lack of understanding of checkpoints in anti-tumor immunity), the period from 2011 to the present can be considered as the beginning of an “age of enlightenment” in melanoma immunotherapy. While much still remains to be understood about why immunotherapy works for some melanoma patients, a roadmap is present for continued progress going forward. The historical Age of Enlightenment is generally considered to be a period in history characterized by a more rational understanding of cause and effect, enabling evidence-based progress. Several key laboratory insights during the late stages of the melanoma “dark ages” were essential for the clinical improvements realized in the melanoma clinic since 2011. For example, the understanding that T cells can specifically recognize melanoma provided the foundation for upcoming discoveries [6]. Subsequent insights into mechanisms of T cell activation provided opportunities to regulate T cell responses and achieve impressive antitumor activity, initially in preclinical models and then in the clinic [7]. A landmark clinical trial initially reported improved survival with ipilimumab in metastatic melanoma [8]. Clinical testing then demonstrated improved outcome with less toxicity with pembrolizumab versus ipilimumab in metastatic melanoma [9]. Most of us in the melanoma community remember well the excitement during the presentation by Dr. Jedd Wolchok at the 2013 ASCO meeting, subsequently reported in the NEJM, of rapid and deep tumor regression in a substantial proportion of metastatic melanoma patients participating in a phase 1 study involving concurrent administration of ipilimumab and nivolumab [10]. Subsequent analysis reported improved OS with combination therapy with nivolumab plus ipilimumab and with nivolumab monotherapy versus ipilimumab monotherapy in patients with previously untreated advanced melanoma [11]. While not powered to compare the two nivolumab arms, combination therapy with ipilimumab and nivolumab resulted in a higher objective response rate than nivolumab alone regardless of the tumor PD-L1 expression level. While descriptive comparisons between the two nivolumab-containing groups suggested better survival with combination ipilimumab and nivolumab therapy than with nivolumab monotherapy in patients with a lower tumor PD-L1 expression level, OS was similar between the nivolumab plus ipilimumab group and the nivolumab monotherapy group among patients with a tumor with a PD-L1 expression level of 1% or more or a PD-L1 expression level of 5% or more [11]. This search for predictive biomarkers of response is key, as grade 3 or 4 toxicity occurred in 59% of patients treated with the combination of ipilimumab plus nivolumab in contrast to occurring in only 21% of patients treated with nivolumab monotherapy [11]. A similar search for biomarkers of adverse events is equally important. If we knew which patients were likely or less likely to experience a grade 3–4 adverse event, that information would help guide patient treatment.

The current report of a 3-year OS rate of 63% for advanced melanoma patients treated in phase 1 dose escalation study CA209–004 (*n* = 53) or in an expansion cohort with the dose and schedule of concurrent ipilimumab and nivolumab now approved for patients with unresectable or metastatic melanoma (*n* = 41) is an impressive accomplishment, especially when considered with the historical perspective of metastatic melanoma outcomes during the recent “dark ages”. One obvious limitation of the current report is the exploratory nature of the OS endpoint in this study and the limitations with cross study comparisons. The candidate PD-L1 biomarker was indeterminant/not evaluable/missing in 48.8% of the expansion cohort patients, and none of the expansion cohort patients had a tumor with documented PD-L1 expression of 5% or greater. There remains a need to identify both predictive and prognostic biomarkers for melanoma patients considering treatment with the combination of ipilimumab and nivolumab. While data about subsequent treatments were not collected, initial responses often occurred off treatment (Figure 2 in the Callahan article). Thus, the potential impact of subsequent treatments on OS cannot be determined with these data. However, the 3-year OS rate of 63% for advanced melanoma is a notable finding. In addition, the report of a low likelihood of significant late adverse events is important for subsequent clinical investigation of treatments in combination with ipilimumab and nivolumab. Further progress in the field is also anticipated with clinical investigation of combination immunotherapy strategies involving anti-PD1 (pembrolizumab or nivolumab) with T-VEC, experimental vaccine strategies, and novel agents.

Laboratory insights from preclinical models have improved survival and have changed the standard of care for metastatic melanoma patients. An example will highlight the importance of continuing this approach. Combining an in situ cancer vaccine with immune checkpoint blockade is an attractive strategy to amplify antitumor immune responses and improve clinical outcome. A potential limitation of this strategy is the possibility of distant untreated tumor sites mediating a suppressive effect on the local and systemic response to in situ vaccination, a process termed “concomitant immune tolerance” [12]. A recent report suggested that tumor-specific Tregs harbored in untreated tumors may pose a challenge to the efficacy of in situ vaccination, and identified potential therapeutic approaches to deplete local Tregs to circumvent this problem [12]. We plan to further study the potential impact of concomitant immune tolerance in a large animal model involving spontaneous canine melanoma to inform a clinical trial in development that includes an in situ cancer vaccine, local radiotherapy, and immune checkpoint blockade.

In conclusion, immune checkpoint blockade can achieve durable responses in many patients with metastatic melanoma, and current treatments can improve survival for many metastatic melanoma patients as well as provide hope for a cure for some of them. Awareness of possible immune-related adverse events is essential following immune checkpoint blockade therapy, and biomarkers of adverse events as well as improved predictive biomarkers of response are needed for current melanoma treatments. There is great enthusiasm to study treatment combinations with immune checkpoint blockade. Our roadmap is clear: transformative insights in the lab will continue to guide progress in the melanoma clinic. While meaningful progress is being made, much more work still needs to be accomplished for patients with metastatic melanoma.

## Abbreviations

DTIC: dacarbazine
FDA: Food and Drug Administration
IL-2: interleukin-2
MAPK: Mitogen-activated protein kinase
OS: Overall survival
T-VEC: Talimogene laherparepvec
